# Dental Fees

**Published:** 1861-03

**Authors:** W. Cahoon

**Affiliations:** Detroit


### DENTAL FEES.

Essay read before the Michigan Dental Association.

BY DR. W. CAHOON, DETROIT.

In this fast age, when dollars and cents are needed to place men in positions in society, it seems right that the dentist should consider well the matter of fees in all its bearings. Men may talk of philanthropy and generosity. It sounds all well; but the maxim is still good, to be just before being generous. Not that a man should not be generous, but that he be allowed to bestow his beneficence as he likes, and not be compelled, because he has chosen a profession, to compromise with every one with whom he has to do. The dentist who is fully prepared, in his profession, to do all that the age and times require of him, has not come to that point without an expenditure of money and labor that none but he who has experienced it can estimate; therefore the dentist should be allowed to charge for his services in proportion to the amount of service rendered, there being such a difference in cases and operation performed, that it seems almost impossible to have any set fees to guide in an estimate until the work is done. Although the patient may inquire beforehand how much will be the charge, let it be distinctly understood that the charges will be made in proportion to the time occupied, and the amount of material used, as no other method can enable the operator to do justice to his patients and himself.

And then, in his estimate of services, let him be conscientious and honorable; and, if he knows he has rendered good service, let him charge a good fee, and in that way he will not only gain the respect and confidence of his patients, but drive the wolf from his own door. Besides, operations performed under considerations like these, will certainly be more valuable to all parties concerned, than if done under a stipulated fee beforehand; as those wanting anything done to their teeth want it done as well as possible, and, in nine cases out of ten, are willing to pay anything within the bounds of reason.

It is true, there will be a great many cases presented, in which there can be an estimate made; but to make it a general rule, would prove ruinous. The dentist, like all other men, is ambitious to make a respectable appearance in society, and to give his family the benefits of an education, and to provide for a rainy day; therefore, he must charge more for his services than the artisan, who can manufacture his wares in large quantities, then throw them into the market for sale; while the dentist has got to do his own work, and wait until he shall have an order, even to do that.

The price charged, however high it may be, by a dentist who is known to do his work well, gives better satisfaction than though he was a second or third rate operator, and charged accordingly.

